# Anti-Photoaging Effect of Plant Extract Fermented with *Lactobacillus buchneri* on CCD-986sk Fibroblasts and HaCaT Keratinocytes

**DOI:** 10.3390/jfb11010003

**Published:** 2020-01-09

**Authors:** Yun-Mi Kang, Chul-Hee Hong, Sa-Haeng Kang, Dong-Seok Seo, Seong-Oh Kim, Hoon-Yeon Lee, Hyeon-Jae Sim, Hyo-Jin An

**Affiliations:** 1 Department of Pharmacology, College of Korean Medicine, Sangji University, Gangwon-do 26339, Korea; yunmi6115@naver.com; 2 Department of Korean Ophthalmology and Otolaryngology and Dermatology, College of Korean Medicine, Sangji University, Wonju, Gangwon 26339, Korea; hong7250@sangji.ac.kr; 3 Department of Oriental Pharmacy, College of Pharmacy and Wonkwang-Oriental Medicines Research Institute, Iksan, Jeonbuk 59338, Korea; rkdtkgod@naver.com; 4 WonNature, Wonkwang University, Iksan, Jeonbuk 54538, Korea; ym5818@naver.com; 5 Research Institute, Wonkwang herb Co., Ltd., Jinan, Jeonbuk 55442, Korea; ilovehanyak@naver.com (S.-O.K.); lovely4034@naver.com (H.-Y.L.); kshold@hanmail.net (H.-J.S.)

**Keywords:** *Lactobacillus buchneri*, photoaging, UVB, metalloproteinases, reactive oxygen species

## Abstract

Ultraviolet (UV) exposure triggers the abnormal production of reactive oxygen (ROS) species and the expression of matrix metalloproteinases (MMPs) that are responsible for photoaging. Probiotics are widely used in healthcare and for immune enhancement. One probiotic, *Lactobacillus buchneri* is found in Kimchi. This study was aimed at assessing the anti-photoaging effect of plant extracts fermented with *L. buchneri* (PELB) to develop functional cosmetics. We investigated the anti-photoaging effect of PELB in a UVB-induced photoaging in vitro model and selected effective extracts using the elastase inhibition assay, ELISA for Type I procollagen and collagenase-1, and quantitative real time PCR. Normal human dermal fibroblasts and epidermal keratinocytes were pre-treated with PELB and exposed to UVB. We found that PELB decreased elastase activity and increased type I collagen expression in a UVB-induced photoaging in vitro model. In addition, PELB greatly reduced collagenase activity and MMP mRNA levels in a UVB-induced photoaging in vitro model. Furthermore, PELB promoted the expression of moisture factor and anti-oxidant enzymes in a UVB-induced photoaging in vitro model. These results indicated that the PELB could be potential candidates for the protective effects against UVB-induced photoaging. Overall, these results suggest that PELB might be useful natural components of cosmetic products.

## 1. Introduction

Skin aging due to chronological aging is caused by decreased skin physiological function and extrinsic factors such as ultraviolet (UV) radiation, stress, reactive oxygen species (ROS), and chemicals. UV sun radiation is considered to be the main extrinsic factor of skin aging, with photoaging being characterized by wrinkles, depigmentation, roughness, loss of moisture, low elasticity, and diverse changes in fibroblast, keratinocytes, and infiltrating neutrophils [1]. UV radiation consist of UVA, UVB, and UVC. UVB (280–320 nm) is mostly responsible for oxidative stress, DNA damage, wrinkle formation, and inflammation in photoaging. ROS induced by UV irradiation mediate diverse cellular functions and alterations, including matrix metalloproteinases (MMPs) secretion and extracellular matrix (ECM) degradation [2]. It is well documented that prolonged UVB exposure induces skin aging, leading to wrinkle formation because of collagen and elastin fiber breakdown along with the inhibition of procollagen synthesis [3]. 

The ECM is comprised of numerous proteins, including collagen and elastin, all of which play a major role in retaining skin elasticity. Collagen is the main structural component of the dermis, and is synthesized from procollagen as a precursor in dermal fibroblasts [4]. Elastic fibers mainly constitute the elastin and form a network associated with collagen [5]. ROS generated by UVB irradiation induce the dermal fibroblasts and epidermal keratinocytes to release MMPs, which accelerate the degradation of key components such as collagen, elastin, and other ECM proteins, thereby leading to skin aging [6,7].

The World Health Organization defines probiotics as “live microorganisms (usually bacteria) that are similar to beneficial microorganisms when administered in an adequate amount and confer a health benefit to the host” [8]. They have received attention for their beneficial effect on digestive health in addition to modulating the immune system against allergic diseases and atopic dermatitis. Recently, their potential roles in preventing photodamage, aging, and skin problems has been suggested [9,10]. Prebiotics are non-digestible dietary ingredients including dietary fibers that are selectively fermented to stimulate the growth of beneficial bacteria and to assist the activity of the probiotics in the intestine [11]. Prebiotics were combined with probiotics to form what is termed as synbiotics, introduced by Gibson and Roberfroid in 1995 [12]. The concept of synbiotics means literally “synergy” that occurred as the prebiotics feed and the probiotics organisms affect the host in improving the therapeutic benefits. Synbiotics have been recognized as an attractive treatment for health maintenance and disease prevention since it beneficially affects the possible survival for probiotics [13]. Studies have highlighted that synbiotics positively affect inflammatory bowel disorder [14], immune function [15], atopic dermatitis [16], and type 2 diabetes [17]. 

*Lactobacillus* is a strain of lactic acid bacteria (LAB) found in the gastrointestinal tract, and these are the most commonly used probiotics in foods. The studies on LAB are usually focused on anti-inflammation and intestinal immune system, with a range of health-promoting effects having been reported for the genus *Lactobacillus* [18,19,20]. Furthermore, fermentation with probiotics potentially increases the number of microorganisms in the diet and can result in new salutary compounds, which can be beneficial to the body [21]. *Lactobacillus buchneri*, derived from Kimchi or cheese, is a heterofermentative bacterial species that produces lactic acid and acetic acid during fermentation and has been reported to improve the storage of food or feed. Recent studies have demonstrated that *L. buchneri* also exert probiotic activities [22,23,24,25]. It was reported that lipoteichoic acid isolated from *L. plantarum* has a photo-protective effect on skin [26], however, the possible effects of *L. buchneri* on skin have not been studied. In the present study, we evaluated the effects of five plant extracts fermented with *L. buchneri* in UVB-induced human dermal fibroblast CCD-986sk cells and human keratinocyte HaCaT cells in vitro photoaging model to develop effective materials for functional anti-wrinkle cosmetics. 

## 2. Materials and Methods

### 2.1. Chemicals and Reagents

Iscove’s modified Dulbecco’s medium (IMDM), Dulbecco’s Modified Eagles medium (DMEM), Dulbecco’s phosphate-buffered saline (DPBS), fetal bovine serum (FBS), penicillin, and streptomycin were purchased from Life Technologies Inc. (Grand Island, NY, USA). 3-(4,5-dimethylthiazol-2-yl)-2,5-diphenyltetrazolium bromide (MTT) and dimethyl sulfoxide (DMSO) were purchased from Junsei Chemical Co., Ltd. (Tokyo, Japan). N-Succinyl-Ala-Ala-Ala-p-nitroanilide (STANA), elastase from porcine pancreas, all-trans retinol, and adenosine were purchased from Sigma Chemical Co. (St. Louis, MO, USA). Procollagen Type I C-peptide (PIP) EIA kit was purchased from Takara Bio Inc. (Kusatsu, Shiga, Japan). Human Total MMP-1 DuoSet ELISA kit was obtained from R&D Systems (Minneapolis, MN, USA).

### 2.2. Preparation of Plant Extract with L. Buchneri (PELB)

The bacterial species were identified using 16S rRNA gene sequence analysis with higher similarity with *L. buchneri* strain JCM 1115 16S ribosomal RNA gene, thus, *L. buchneri* was selected as a candidate. Among Kimchi-derived lactic acid bacteria (genus *Lactobacillus*), *L. buchneri* was selected. *L. buchneri* and the plants (*Triticum aestivum* Leaf, *Avena sativa* L., *Helianthus tuberosus* L., *Glycine max* Merr. with inner color-greenish, and *Smallanthus sonchifolius*) are provided by Wonkwang herb Co., Ltd. (Jinan, Jeollabuk-do, Korea). Using *L. buchneri* and five plants, lactic acid bacterial fermented extracts were produced. Extracts of five plants fermented with *L. buchneri* were obtained from Wonkwang University, Wonnature (Iksan, Jeollabuk-do, Korea). Briefly, after 10 min of sterilization at 100 °C, probiotics were inoculated into natural products, then put into an agitated extractor, followed by the production of lactic acid bacteria fermented extract. After fermentation, 50% ethanol was added and fermentation was continued for 24 h. After filtering the extracts, the extracts were concentrated using the decompression concentrator, freeze-dried, and the lactic acid bacteria fermented extract powder was extracted. The list of PELB is shown in Table 1. The freeze-dried samples were dissolved in distilled water at the final concentration of 50 mg/mL for bioassays.

### 2.3. Cell Culture 

CCD-986sk fibroblasts was purchased from Korea Cell Line Bank (KCLB, Seoul, Korea). The cells were incubated at 37 °C in IMDM supplemented with 10% FBS, penicillin (100 U/mL), and streptomycin (100 μg/mL) in a humidified atmosphere with 5% CO_2_. HaCaT keratinocytes were provided by Professor Jae-Young Um (Kyung Hee University, Korea), and were incubated at 37 °C in DMEM supplemented with 10% FBS, penicillin (100 U/mL), and streptomycin (100 μg/mL) in a humidified atmosphere with 5% CO_2_.

### 2.4. MTT Assay for Cell Viability 

Cell viability was assessed using the MTT assay. Briefly, cells were treated with each of the PELB and incubated for 24 h, followed by incubation with MTT solution (5 mg/mL) for 4 h at 37 °C. After discarding the supernatant, the insoluble formazan product was dissolved in DMSO. Cell viability was measured at 540 nm using a microplate reader (Titertek Multiskan, Huntsville, AL, USA).

### 2.5. UV Irradiation 

As a UVB source, a UVB lamp (BLX crosslinker, Vilber BIO-LINK, France), having an emission spectrum of 280–370 nm and a peak at 312 nm was used. The cells were starved in serum-free medium for 24 h and treated with PELB in serum-free medium for 24 h. Before UV irradiation, the cells were washed twice with DPBS and submerged in a minimum volume of DBPS, followed by exposure to UVB light at a rate of 20 or 50 mJ/cm^2^ using a UVB lamp. After UV irradiation, the cells were washed with DPBS and cultured for 6 or 24 h in serum-free medium. 

### 2.6. Elastase Inhibition Assay 

Elastase inhibition activity was determined according to a previously published method [27] with some modifications. 50 µL of 50 mM Tris-HCl buffer (pH 8.0), 50 µL of 0.4 units/mL elastase from porcine pancreas, and 50 µL of PELB were mixed and incubated for 30 min at 37 °C. Then, 100 µL of 1 mM STANA was added to the cells, which were then incubated for another 30 min. The elastase inhibition rate was measured using a microplate reader at 410 nm and calculated as follows: Inhibition rate (%) = [1 − (Absorbance of sample/Absorbance of control)] × 100. 

### 2.7. Type I Procollagen Synthesis Measurement 

CCD-986sk fibroblasts were seeded at a density of 1 × 10^5^ cells per well, starved in serum-free media for 24 h, and treated with PELB at 200 and 400 μg/mL for 24 h at 37 °C in humidified air with 5% CO_2_. The cells were exposed to UVB light at a rate of 20 mJ/cm^2^ using UVB lamp and incubated for 24 h in the serum-free media. Harvested cell supernatants were used for the experiments. Collagen assay was performed using an ELISA kit in accordance with the manufacturer’s protocol.

### 2.8. Collagenase Inhibition Assay 

CCD-986sk fibroblasts or HaCaT keratinocytes were seeded at a density of 1 × 10^5^ cells per well, starved in serum free media for 24 h, and treated with PELB at the indicated concentration for 24 h at 37 °C in humidified air with 5% CO_2_. The cells were exposed to UVB light at a rate of 20 or 50 mJ/cm^2^ using UVB lamp and incubated for 24 h in the serum-free media. Tumor necrosis factor (TNF)-α (10 ng/mL) was added after UVB irradiation in order to enhance the activity of MMP-1. Harvested cell supernatants were used in the experiments. MMP-1 production was measured using an ELISA kit in accordance with the manufacturer’s protocol.

### 2.9. Quantitative Real Time Polymerase Chain Reaction (qRT-PCR) 

Total RNA was isolated from the cells using an Easy Blue kit (Intron Biotechnology, Inc., Seoul, Korea) according to the manufacturer’s protocol. Total RNA was quantified using an Epoch micro-volume spectrophotometer system (BioTek Instruments, Inc., Winooski, VT, USA). cDNA was synthesized from the isolated total RNA (2 μg), d(T)16 primer, and Avian Myeloblastosis Virus reverse transcriptase with genomic DNA remover. The relative gene expression was quantified using RT-qPCR analysis (Real Time PCR System 7500; Applied Biosystems; Thermo Fisher Scientific, Inc., Waltham, MA, USA) with SYBR Premix Ex Taq. The PCR cycling conditions were as follows: 10 min at 95 °C; 40 cycles of 5 s at 95 °C and 45 s at 60 °C; and a final melting curve of 15 s at 95 °C, 1 min at 60 °C, and 15 s at 95 °C. The oligonucleotide primers were as follows: Human MMP-1, forward 5′-gatgaagcagcccagatgtg-3′ and reverse 5′-gcttgaccctcagagacctt-3′; MMP-9, forward 5′-gagttcccggagtgagttga-3′ and reverse 5′-aaaggtgagaagagagggcc-3′; HAS1, forward 5′-ctcagtttccctcctctgca-3′ and reverse 5′-gaggagaaagcaggaccctt-3′; CAT, forward 5′-gagcctacgtcctgagtctc-3′ and reverse 5′-atcccggatgccatagtcag-3′; SOD, forward 5′-ggagacttgggcaatgtgac-3′ and reverse 5′-cacaagccaaacgacttcca-3′; GAPDH, forward 5′-aattccatcggcaccgtcaag-3′ and reverse 5′-atcgccccacttgattttgg-3′. Fold changes of gene expression were calculated using the comparative quantification cycle (Cq) method. The Cq values of target genes MMP-1, MMP-9, HAS1, CAT, and SOD were normalized to that of GAPDH using the ABI gene express 2.0 program (Applied Biosystems; Thermo Fisher Scientific, Inc., Waltham, MA, USA). 

### 2.10. Statistical Analysis 

Data are expressed as the mean ± standard deviation (SD) for triplicate experiments. Statistically significant values were compared using ANOVA and Dunnett’s post hoc test, and *p*-values < 0.05 were considered statistically significant. Statistical analysis was performed using SPSS statistical analysis software (version 19.0, IBM SPSS, Armonk, NY, USA).

## 3. Results

### 3.1. Effects of PELB on Cell Viability 

MTT assays were performed to confirm the effect of PELB on cell viability in CCD-986sk fibroblasts and HaCaT keratinocytes. As shown in Table 2, treatment with TAB, ASB, HTB, GMB, and SSB in the concentration range from 0 to 400 μg/mL for 24 h had no effect on the cell viability in CCD-986sk fibroblasts. On the other hand, the 5 PELB exhibited no cytotoxicity and remarkable cell proliferation in HaCaT keratinocytes. The viability of TAB, ASB, HTB, and GMB-treated cells increased by 1.6, 1.3, 1.2, and 1.5 times at the highest concentration compared to control, respectively (Table 3). Accordingly, we investigated the anti-photoaging effects of the extracts’ concentration < 400 μg/mL in both cell types.

### 3.2. Effects of PELB on Elastase Activity

To examine the effects of PELB on elastase effect, we determined the porcine pancreas elastase activity upon treatment with the different PELB. When the five PELB were treated with the specific concentration, they showed high elastase inhibition activity in TAB, HTB, and GMB in the corresponding order. Additionally, TAB at low concentration (6.25 to 12.5 g/mL) and HTB at high concentration (100 to 200 g/mL) showed similar or more active elastase inhibition activity than that of retinol and adenosine that was used as positive control (Table 4).

### 3.3. Effects of PELB on Type I Procollagen Synthesis in UVB-Irradiated CCD-986sk Cells

**Figure 1:**
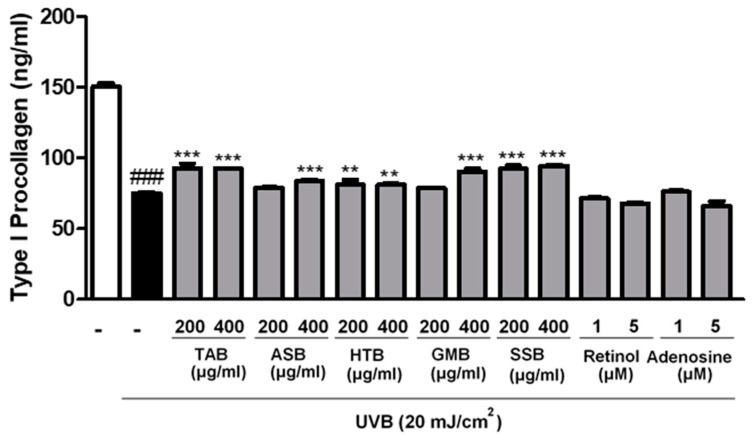
PELB promoted type I procollagen synthesis in UVB-irradiated CCD-986sk fibroblasts. CCD-986sk fibroblasts were pre-treated with the indicated concentration for 24h in serum-free media, and after UVB (20 mJ/cm^2^) irradiation, type 1 procollagen was measured from the supernatant. Retinol and adenosine were used as positive control. ^###^ *p* < 0.001 versus the control group; ** *p* < 0.01, and *** *p* < 0.001 versus the UVB-irradiated group.

Type I collagen is a major component of the skin structural protein, produced by fibroblasts and has functional role in maintaining the integrity and tension of dermis [28]. Procollagen is a precursor of collagen, the final synthesized form. We measured the amount of type I procollagen, which is the most abundant collagen in skin dermis. To examine the effect of PELB on collagen synthesis in skin, CCD-986sk fibroblasts were pretreated with TAB, ASB, HTB, GMB, and SSB at 200 and 400 μg/mL for 24 h and exposed to UVB. UVB irradiation decreased type I procollagen level to 75.26 ng/mL in comparison to non-irradiated control (150.33 ng/mL). However, the levels of type I procollagen were significantly increased by the pretreatment of five PELB compared to UVB-irradiated group. The positive control, retinol (1 and 5 μM) and adenosine (1 and 5 μM), did not show any significance for the recovery of type I procollagen (Figure 1).

### 3.4. Effects of PELB on Collagenase Production in UVB-Irradiated HaCaT Keratinocytes

**Figure 2:**
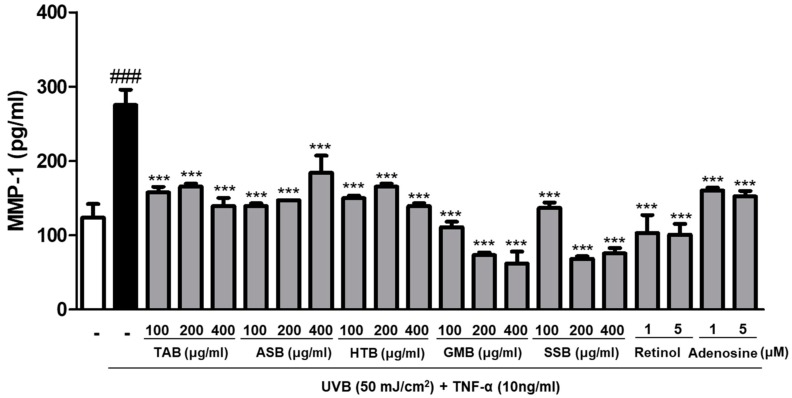
PELB inhibited collagenase-1 (MMP-1) production in UVB-irradiated HaCaT keratinocytes. HaCaT keratinocytes were pre-treated with the indicated concentration for 24h in serum-free media, and incubated for 24 h followed by UVB (50 mJ/cm^2^) irradiation treated with TNF-α (10 ng/mL), collagenase-1 (MMP-1) was measured from the supernatant. Retinol (1 and 5 μM) and adenosine (1 and 5 μM) were used as positive control. ^###^ *p* < 0.001 versus the control group; *** *p* < 0.001 versus the UVB-irradiated group.

Collagenases are the major collagenolytic enzymes responsible for collagen damage in UV-irradiated human skin [29]. To evaluate the collagenase inhibition effect of PELB, we measured the level of collagenase 1, known as MMP-1, associated with collagen degradation in skin dermis. Compared with the non-irradiated group, MMP-1 levels in HaCaT keratinocytes were markedly elevated to 275.195 pg/mL upon UVB irradiation. When the cells were pretreated with various concentrations of PELB, the trend was reversed. The pretreatment with TAB, ASB, HTB, GMB, and SSB significantly inhibited the production of MMP-1 by 49.39%, 33.04%, 49.40%, 77.44%, and 72.52% at a concentration 400 μg/mL, respectively, in UVB-irradiated HaCaT keratinocytes. PELB showed similar or better collagenase inhibition than that of retinol or adenosine that were used as positive controls (Figure 2). 

### 3.5. Effects of PELB on the mRNA Expression of MMPs in UVB-Irradiated HaCaT Keratinocytes

**Figure 3:**
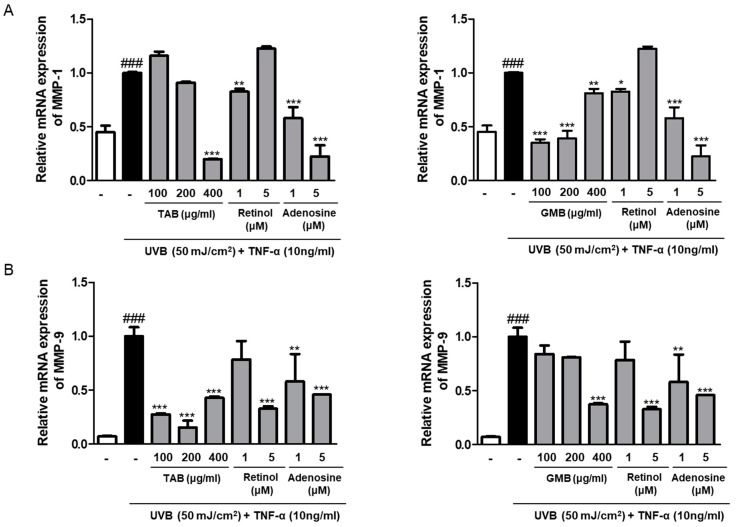
TAB and GMB suppressed mRNA expression of MMPs in UVB-irradiated HaCaT keratinocytes. HaCaT keratinocytes were pre-treated with the indicated concentration for 24 h in serum-free media, and incubated for 6 h followed by UVB (50 mJ/cm^2^) irradiation treated with TNF-α (10 ng/mL), (**A**) MMP-1 and (**B**) MMP-9 mRNA levels were measured by qRT-PCR. Retinol (1 and 5 μM) and adenosine (1 and 5 μM) were used as positive control. ^###^ *p* < 0.001 versus the control group; * *p* < 0.05, ** *p* < 0.01, and *** *p* < 0.001 versus the UVB-irradiated group. MMP-1, matrix metalloproteinase-1; MMP-9; matrix metalloproteinase-9.

From the screening results, we selected TAB and GMB treated groups that showed a relatively good anti-aging effect. Next, we evaluated the effect of PELB on the mRNA expression of MMPs in UVB-irradiated HaCaT keratinocytes. UVB irradiation significantly increased the mRNA levels of MMP-1 and MMP-9, whereas TAB and GMB pretreatment inhibited the mRNA expression of MMPs in UVB-irradiated HaCaT keratinocytes. TAB, at a concentration of 400 μg/mL, lowered the expression of UVB-induced MMP-1 by 80.04% and GMB, at a concentration of 100 μg/mL, decreased MMP-1 expression by 64.97%, compared to the UVB-irradiation (Figure 3A). In addition, UVB caused a markedly increased MMP-9 mRNA expression. However, treatment with TAB and GMB significantly downregulated UVB-induced MMP-9 expression by 84.70% at 200 μg/mL and 62.69% at 400 μg/mL, respectively (Figure 3B). 

### 3.6. Effects of PELB on the mRNA Expression of Moisture and Antioxidant Enzymes in UVB-Irradiated HaCaT Keratinocytes

**Figure 4:**
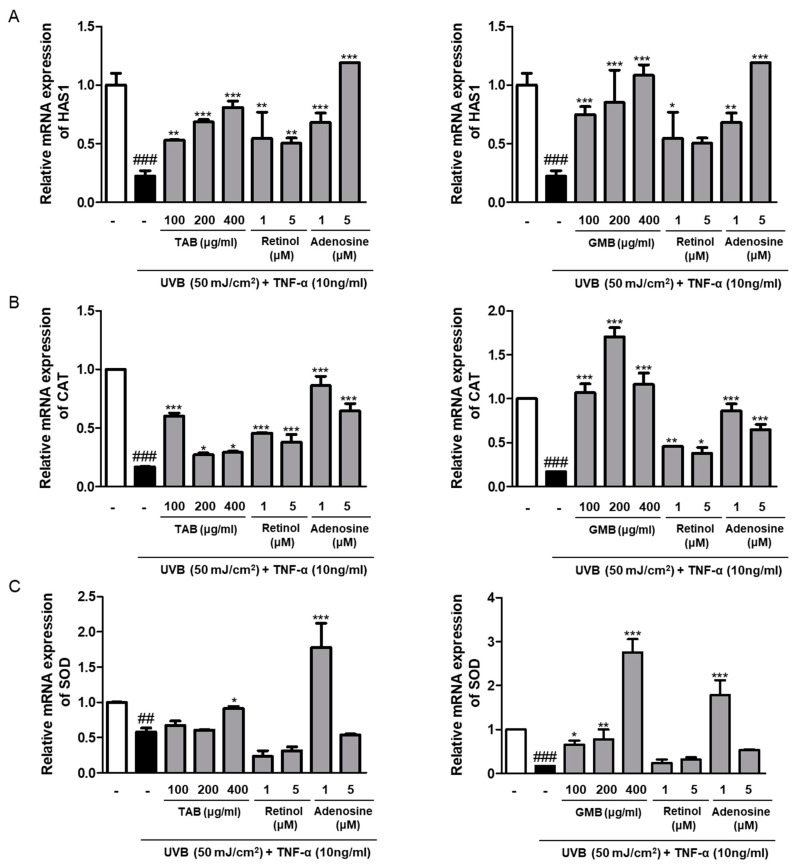
TAB and GMB recovered mRNA expression of moisture and antioxidant enzymes in UVB-irradiated HaCaT keratinocytes. HaCaT keratinocytes were pre-treated with the indicated concentration for 24 h in serum-free media, and incubated for 6 h followed by UVB (50 mJ/cm^2^) irradiation treated with TNF-α (10 ng/mL), (**A**) HAS1, (**B**) CAT, and (**C**) SOD mRNA levels were measured by qRT-PCR. Retinol (1 and 5 μM) and adenosine (1 and 5 μM) were used as positive control. ^##^ *p* < 0.01, ^###^ *p* < 0.001 versus the control group; * *p* < 0.05, ** *p* < 0.01, and *** *p* < 0.001 versus UVB-irradiated group. HAS1, hyaluronic acid synthase 1; CAT, catalase; SOD, superoxide dismutase.

Skin wrinkles are closely related to skin moisture that can provide the firmness and bounciness in skin, and hyaluronic acid is known as the moisturizing factor that has a unique capacity to bind and retain water molecules [30]. We evaluated the effect of TAB and GMB on the expression of hyaluronic acid synthase 1 (*HAS 1*) released from keratinocytes. As expected, the pretreatment of TAB and GMB increased the mRNA expression of *HAS 1*, which were decreased upon UVB irradiation. Specifically, GMB dose-dependently promoted the expression of *HAS 1* more than 3–4 times compared to UVB-irradiated group (Figure 4A). UVB irradiation resulted in ROS generation, which induced the oxidation of DNA, RNA, and protein, including cellular components. The mRNA expression of anti-oxidant enzymes such as catalase (CAT), and superoxide dismutase (SOD) were reduced upon UV exposure, whereas the mRNA expressions were significantly increased by TAB and GMB in the UVB-irradiated HaCaT keratinocytes (Figure 4B,C). 

## 4. Discussion 

Photoaging is a noticeable premature aging of the skin caused by prolonged and repetitive exposure to UV, and its concerns are worldwide because it is linked to a series of physiologic and pathological processes. In the present study, we focused on the characteristics of photo-aged skin and evaluated the anti-photoaging effect of plant extracts fermented with *L. buchneri* according to the Korean Ministry of Food and Drug Safety guidelines for the evaluation of the efficacy of functional anti-wrinkle cosmetics.

Elastase is one of the proteases that is mainly accountable for the breakdown of elastin as well as collagen, fibronectin, and other ECM proteins in dermal skin [31]. Our screening results on comparing the efficacies of the five plant extracts fermented with *L. buchneri* showed that TAB, HTB, and GMB had an inhibitory effect on the elastase activity in comparison to positive controls (Table 4). On the other hand, all of the five plant extracts fermented with *L. buchneri* significantly decreased the expression of type I procollagen upon UVB irradiation (Figure 1). Such effects of the plant extracts fermented with *L. buchneri* were not related to cytotoxicity, because they had no effect on cell viability. Instead, the plant extracts fermented with *L. buchneri* were safe as they induced cell proliferation (Table 2 and Table 3). 

UV irradiation induces the expression of MMPs, which break down collagen and other ECM proteins and participate in structurally diverse and destructive processes in the skin. MMPs can be classified as collagenases, gelatinases, matrilysins, stromelysins, and membraned-type MMPs, depending on their function [32]. MMP-1, one of the collagenase subfamilies of MMPs, degrades type I collagen, which are further hydrolyzed by gelatinases, MMP-9 [33]. Therefore, we evaluated whether plant extracts fermented with *L. buchneri* affect the expression of MMPs. The five plant extracts fermented with *L. buchneri* remarkably decreased UV-induced MMP-1 production (Figure 2), and the selected plant extracts (TAB and GMB) reduced MMP-1 and MMP-9 mRNA levels (Figure 3). UVB irradiation remarkably induced the expression of MMP-9 compared to the expression of MMP-1 when they are compared to normal group, and TAB significantly attenuated the expression of MMP-9 than MMP-1. Thus, it is necessary to study whether the antiphotoaging effect is more specific to the MMP-9 signaling pathway or there is a difference in the mechanisms of regulation with MMPs. Our data indicated that MMPs are the major factor for UVB-induced photoaging and plant extracts fermented with *L. buchneri* can prevent UVB-induced skin damage by inhibiting the mRNA expression and production of collagenase, resulting in increased type I collagen production. 

Hyaluronic acid (HA) is a component of skin and is well known as an important molecule involved in skin moisture and firmness in association with its water retention activity. HA is produced and released by fibroblasts, keratinocytes, and chondrocytes. Hyaluronic acid synthases (HAS1, HAS2, and HAS3) are the enzymes belonging to a class of integral membrane proteins involved in hyaluronic acid synthesis [34]. Our results showed that plant extracts fermented with *L. buchneri* can prevent the UVB-induced downregulation of HAS1 mRNA expression in human epidermal HaCaT keratinocytes (Figure 4). These results supported the anti-photoaging effects of TAB and GMB in this study.

UVB irradiation results in ROS generation, which is considered a potent inducer of MMP expression and skin aging. This indicates that the prevention of ROS is essential for reducing photoaging by blocking MMP production. Oxidative stress caused by UV reduces the expression of anti-oxidant enzymes, such as catalase, SOD, and glutathione peroxidase, which protect the skin epidermis and dermis [35]. The mRNA levels of CAT and SOD were remarkably increased by the pretreatment of GMB, which is more effective even in positive controls. The pretreatment of TAB also increased the levels of CAT and SOD; however, there was no significant difference. These data indicated that TMB and GMB have an anti-aging effect, activating antioxidant enzymes, and suggested the possibility of an ROS scavenging ability of TAB and GMB, revealing them as promising photo-protective agents. Considering all the data, it can be hypothesized that the anti-photoaging effect originated from γ-Aminobutyric acid (GABA), β-glucan in *Triticum aestivum* Leaf, isoflavone, and the other flavonoids in *Glycine max* Merr. with inner greenish-color. These components are well known for their beneficial effects on improving skin elasticity and reducing wrinkles in skin. Oxidative stress induced by ROS triggers the activation of the mitogen-activated protein kinase (MAPK), which affects the regulation of transcription factor AP-1 activity. Moreover, the activation of the transcription factor NF-κB leads to the expression of pro-inflammatory cytokines, such as COX-2, IL-6, and MMPs [36]. Therefore, further studies are necessary to understand and elucidate the molecular mechanisms related to the anti-photoaging effect of TAB and GMB and their active compounds.

## 5. Conclusions

In conclusion, these results suggest that the anti-wrinkle and anti-photoaging effects of PELB result from the enhancement of type I procollagen synthesis and the inhibition of elastase activity and the expression of UVB-induced MMPs by alleviating the effect of ROS in a UVB-induced photoaging in vitro model. Therefore, plant extracts fermented with *L. buchneri*, particularly TAB and GMB, might have a potential role as anti-wrinkle cosmetic agents.

## Figures and Tables

**Table 1:** List of 5 plant extracts with *Lactobacillus buchneri.*

Number	Scientific Name	Abbreviation
1	Extract of *Triticum aestivum* Leaf fermented with *Lactobacillus buchneri*	TAB
2	Extract of *Avena sativa* L. fermented with *Lactobacillus buchneri*	ASB
3	Extract of *Helianthus tuberosus* L. fermented with *Lactobacillus buchneri*	HTB
4	Extract of *Glycine max* Merr. with inner color-greenish fermented with *Lactobacillus buchneri*	GMB
5	Extract of *Smallanthus sonchifolius* fermented with *Lactobacillus buchneri*	SSB

**Table 2:** Effect on cell viability (%) of five plant extracts with *L. buchneri* on CCD-986sk fibroblasts.

Concentration (μg/mL)	Cell Viability (%)				
	TAB	ASB	HTB	GMB	SSB
0	100.00 ± 2.80	100.00 ± 2.80	100 ± 2.80	100 ± 2.80	100 ± 2.80
6.25	96.87 ± 3.13	98.52 ± 2.21	95.92 ± 1.04	100.26 ± 2.79	97.01 ± 1.29
12.5	96.87 ± 3.55	95.83 ± 2.11	98.96 ± 2.22	99.56 ± 2.39	100.49 ± 2.43
25	99.57 ± 2.27	97.57 ± 2.81	97.13 ± 1.53	99.83 ± 3.42	97.10 ± 6.01
50	99.39 ± 3.77	97.91 ± 0.84	96.00 ± 1.70	100.09 ± 1.19	97.36 ± 3.03
100	100.70 ± 4.70	98.87 ± 1.57	97.13 ± 0.30	101.56 ± 0.75	95.88 ± 5.91
200	102.17 ± 3.01	99.30 ± 1.45	98.52 ± 0.69	101.91 ± 1.38	98.40 ± 2.74
400	101.91 ± 1.84	100.17 ± 1.29	100.35 ± 4.05	101.48 ± 2.79	97.10 ± 5.82

**Table 3:** Effect on cell viability (%) of five plant extracts with *L. buchneri* on HaCaT keratinocytes.

Concentration (μg/mL)	Cell Viability (%)				
	TAB	ASB	HTB	GMB	SSB
0	100.00 ± 5.20	100.00 ± 5.20	100.00 ± 5.20	100.00 ± 5.20	100.00 ± 5.20
6.25	98.51 ± 6.43	102.88 ± 4.37	105.22 ± 2.78	103.06 ± 5.58	103.04 ± 2.49
12.5	100.94 ± 1.98	109.41 ± 2.12	107.80 ± 4.49	105.16 ± 4.88	100.97 ± 4.14
25	111.01 ± 5.40	108.31 ± 5.44	105.55 ± 2.77	104.63 ± 0.84	94.57 ± 1.65
50	112.53 ± 7.18 *	110.86 ± 3.26	102.79 ± 4.68	118.83 ± 0.84 ***	99.15 ± 2.59
100	130.13 ± 3.97 ***	112.18 ± 3.80 *	105.64 ± 4.10	114.20 ± 2.89 ***	103.94 ± 2.20
200	160.24 ± 5.86 ***	111.35 ± 7.95 *	115.44 ± 3.44 ***	132.13 ± 2.51 ***	102.28 ± 1.92
400	159.50 ± 1.37 ***	126.46 ± 2.92 ***	118.46 ± 0.84 ***	146.48 ± 8.25 ***	107.54 ± 1.85 *

* *p* < 0.05, and *** *p* < 0.001 versus non-treated group.

**Table 4:** Elastase inhibition activity (%) of five plant extracts with *L. buchneri.*

Concentration (μg/mL)	Elastase Inhibition Ratio (%) *					Concentration (μM)	Elastase inhibition Ratio (%)	
	TAB	ASB	HTB	GMB	SSB		Retinol	Adenosine
6.25	16.49 ± 2.29	−0.63 ± 4.59	5.95 ± 6.63	3.95 ± 4.41	2.62 ± 1.92	1.5625	6.32 ± 2.54	16.55 ± 1.83
12.5	11.39 ± 5.78	−0.75 ± 6.37	9.89 ± 4.54	4.36 ± 1.14	0.09 ± 1.05	3.125	13.16 ± 3.09	4.37 ± 4.43
25	3.43 ± 3.33	−2.12 ± 3.28	8.82 ± 7.57	2.37 ± 7.17	−1.68 ± 3.35	6.25	6.90 ± 2.09	8.08 ± 1.46
50	7.34 ± 5.65	0.80 ± 3.53	6.17 ± 5.76	3.11 ± 9.31	−4.18 ± 2.39	12.5	13.42 ± 1.22	2.98 ± 2.09
100	9.94 ± 6.38	1.26 ± 3.39	10.20 ± 2.32	0.22 ± 2.22	−1.35 ± 3.93	25	10.09 ± 1.09	11.10 ± 3.94
200	1.91 ± 0.37	−1.36 ± 4.02	14.04 ± 9.53	6.66 ± 3.34	−4.54 ± 3.49	50	14.87 ± 3.80	12.87 ± 1.00
400	−1.68± 2.37	1.21 ± 6.02	4.83 ± 2.48	5.22 ± 8.88	−1.87 ± 3.20	100	1.01 ± 2.24	9.94 ± 1.06

Target porcine pancreas elastase activity 0.4 U/mL. * Elastase Inhibition Ratio (%) = [1 − (Absorbance of sample/Absorbance of control)] × 100 (Mean ± SD).

## Abbreviations

PELB	Plant extracts fermented with *L. Buchneri*
UV	Ultraviolet
ROS	Reactive oxygen species
MMPs	Matrix metalloproteinases
ECM	Extracellular matrix
HAS 1	Hyaluronic acid synthase 1
CAT	Catalase
SOD	Superoxide dismutase

## References

[1] Kim M.J., Woo S.W., Kim M.S., Park J.E., Hwang J.K. (2014). Anti-photoaging effect of aaptamine in UVB-irradiated human dermal fibroblasts and epidermal keratinocytes. J. Asian Nat. Prod. Res..

[2] Sun Z., Park S.Y., Hwang E., Zhang M., Seo S.A., Lin P., Yi T.H. (2017). Thymus vulgaris alleviates UVB irradiation induced skin damage via inhibition of MAPK/AP-1 and activation of Nrf2-ARE antioxidant system. J. Cell. Mol. Med..

[3] Park E.K., Lee H.J., Lee H., Kim J.H., Hwang J., Koo J.I., Kim S.H. (2018). The Anti-Wrinkle Mechanism of Melatonin in UVB Treated HaCaT Keratinocytes and Hairless Mice via Inhibition of ROS and Sonic Hedgehog Mediated Inflammatory Proteins. Int. J. Mol. Sci..

[4] Park B., Hwang E., Seo S.A., Cho J.G., Yang J.E., Yi T.H. (2018). Eucalyptus globulus extract protects against UVB-induced photoaging by enhancing collagen synthesis via regulation of TGF-beta/Smad signals and attenuation of AP-1. Arch. Biochem. Biophys..

[5] Azmi N., Hashim P., Hashim D.M., Halimoon N., Majid N.M. (2014). Anti-elastase, anti-tyrosinase and matrix metalloproteinase-1 inhibitory activity of earthworm extracts as potential new anti-aging agent. Asian Pac. J. Trop. Biomed..

[6] Xuan S.H., Park Y.M., Ha J.H., Jeong Y.J., Park S.N. (2017). The effect of dehydroglyasperin C on UVB-mediated MMPs expression in human HaCaT cells. Pharmacol. Rep..

[7] Pham Q.L., Jang H.J., Kim K.B. (2017). Antiwrinkle effect of fermented black ginseng on human fibroblasts. Int. J. Mol. Med..

[8] FAO/WHO (2001). Evaluation of Health and Nutritional Properties of Powder Milk and Live Lactic Acid Bacteria.

[9] Sharma D., Kober M.M., Bowe W.P. (2016). Anti-Aging Effects of Probiotics. J. Drugs Dermatol..

[10] Im A.R., Kim H.S., Hyun J.W., Chae S. (2016). Potential for tyndalized Lactobacillus acidophilus as an effective component in moisturizing skin and anti-wrinkle products. Exp. Ther. Med..

[11] Singla V., Chakkaravarthi S. (2017). Applications of prebiotics in food industry: A review. Food Sci. Technol. Int..

[12] Gibson G.R., Roberfroid M.B. (1995). Dietary modulation of the human colonic microbiota: Introducing the concept of prebiotics. J. Nutr..

[13] Pandey K.R., Naik S.R., Vakil B.V. (2015). Probiotics, prebiotics and synbiotics—A review. J. Food Sci. Technol..

[14] Pena A.S. (2007). Intestinal flora, probiotics, prebiotics, symbiotics and novel foods. Rev. Esp. Enferm. Dig..

[15] Frei R., Akdis M., O’Mahony L. (2015). Prebiotics, probiotics, synbiotics, and the immune system: Experimental data and clinical evidence. Curr. Opin. Gastroenterol..

[16] Foolad N., Armstrong A.W. (2014). Prebiotics and probiotics: The prevention and reduction in severity of atopic dermatitis in children. Benef. Microbes.

[17] Yoo J.Y., Kim S.S. (2016). Probiotics and Prebiotics: Present Status and Future Perspectives on Metabolic Disorders. Nutrients.

[18] Lee J., Yang W., Hostetler A., Schultz N., Suckow M.A., Stewart K.L., Kim D.D., Kim H.S. (2016). Characterization of the anti-inflammatory Lactobacillus reuteri BM36301 and its probiotic benefits on aged mice. BMC Microbiol..

[19] Rong J., Shan C., Liu S., Zheng H., Liu C., Liu M., Jin F., Wang L. (2017). Skin resistance to UVB-induced oxidative stress and hyperpigmentation by the topical use of Lactobacillus helveticus NS8-fermented milk supernatant. J. Appl. Microbiol..

[20] Chen Y.H., Wu C.S., Chao Y.H., Lin C.C., Tsai H.Y., Li Y.R., Chen Y.Z., Tsai W.H., Chen Y.K. (2017). Lactobacillus pentosus GMNL-77 inhibits skin lesions in imiquimod-induced psoriasis-like mice. J. Food Drug Anal..

[21] Parvez S., Malik K.A., Ah Kang S., Kim H.Y. (2006). Probiotics and their fermented food products are beneficial for health. J. Appl. Microbiol..

[22] Cho Y.R., Chang J.Y., Chang H.C. (2007). Production of gamma-aminobutyric acid (GABA) by Lactobacillus buchneri isolated from kimchi and its neuroprotective effect on neuronal cells. J. Microbiol. Biotechnol..

[23] Holzer M., Mayrhuber E., Danner H., Braun R. (2003). The role of Lactobacillus buchneri in forage preservation. Trends Biotechnol..

[24] Zeng X.Q., Pan D.D., Guo Y.X. (2010). The probiotic properties of Lactobacillus buchneri P2. J. Appl. Microbiol..

[25] Zhou Y., Ni X., Wen B., Duan L., Sun H., Yang M., Zou F., Lin Y., Liu Q., Zeng Y. (2018). Appropriate dose of Lactobacillus buchneri supplement improves intestinal microbiota and prevents diarrhoea in weaning Rex rabbits. Benef. Microbes.

[26] Hong Y.F., Lee H., Jung B.J., Jang S., Chung D.K., Kim H. (2015). Lipoteichoic acid isolated from Lactobacillus plantarum down-regulates UV-induced MMP-1 expression and up-regulates type I procollagen through the inhibition of reactive oxygen species generation. Mol. Immunol..

[27] Ko R.K., Kim G.O., Hyun C.G., Jung D.S., Lee N.H. (2011). Compounds with tyrosinase inhibition, elastase inhibition and DPPH radical scavenging activities from the branches of Distylium racemosum Sieb. et Zucc. Phytother. Res..

[28] Tracy L.E., Minasian R.A., Caterson E.J. (2016). Extracellular Matrix and Dermal Fibroblast Function in the Healing Wound. Adv. Wound Care (New Rochelle).

[29] Brennan M., Bhatti H., Nerusu K.C., Bhagavathula N., Kang S., Fisher G.J., Varani J., Voorhees J.J. (2003). Matrix metalloproteinase-1 is the major collagenolytic enzyme responsible for collagen damage in UV-irradiated human skin. Photochem. Photobiol..

[30] Li W.H., Wong H.K., Serrano J., Randhawa M., Kaur S., Southall M.D., Parsa R. (2017). Topical stabilized retinol treatment induces the expression of HAS genes and HA production in human skin in vitro and in vivo. Arch. Dermatol. Res..

[31] Thring T.S., Hili P., Naughton D.P. (2009). Anti-collagenase, anti-elastase and anti-oxidant activities of extracts from 21 plants. BMC Complement. Altern. Med..

[32] Nagase H., Visse R., Murphy G. (2006). Structure and function of matrix metalloproteinases and TIMPs. Cardiovasc. Res..

[33] Pittayapruek P., Meephansan J., Prapapan O., Komine M., Ohtsuki M. (2016). Role of Matrix Metalloproteinases in Photoaging and Photocarcinogenesis. Int. J. Mol. Sci..

[34] Park H.J., Cho J.H., Hong S.H., Kim D.H., Jung H.Y., Kang I.K., Cho Y.J. (2018). Whitening and anti-wrinkle activities of ferulic acid isolated from Tetragonia tetragonioides in B16F10 melanoma and CCD-986sk fibroblast cells. J. Nat. Med..

[35] Rittie L., Fisher G.J. (2002). UV-light-induced signal cascades and skin aging. Ageing Res. Rev..

[36] Karthikeyan R., Kanimozhi G., Prasad N.R., Agilan B., Ganesan M., Mohana S., Srithar G. (2016). 7-Hydroxycoumarin prevents UVB-induced activation of NF-kappaB and subsequent overexpression of matrix metalloproteinases and inflammatory markers in human dermal fibroblast cells. J. Photochem. Photobiol. B.

